# Evaluation of the correlation of vasculogenic mimicry, ALDH1, KiSS-1, and MACC1 in the prediction of metastasis and prognosis in ovarian carcinoma

**DOI:** 10.1186/s13000-017-0612-9

**Published:** 2017-03-02

**Authors:** Lan Yu, Bo Zhu, Shiwu Wu, Lei Zhou, Wenqing Song, Xiaomeng Gong, Danna Wang

**Affiliations:** Department of Pathology, the First Affiliated Hospital of Bengbu Medical College, Bengbu Medical College, No.287, Changhuai Road, Bengbu, 233003 Anhui Province China

**Keywords:** Epithelial ovarian carcinoma, VM, ALDH1, KiSS-1, MACC1, Prognosis

## Abstract

**Background:**

Recurrence and metastasis are the usual manifestations of treatment failure of epithelial ovarian carcinoma (EOC). Vasculogenic mimicry (VM; blood supply development often seen in highly aggressive cancers), aldehyde dehydrogenase 1 (ALDH1, cancer stem cell biomarker), KiSS-1 (suppressor of tumor metastasis), and metastasis associated in colon cancer-1 (MACC1) are all useful predictive factors for metastasis and prognosis in various cancers. In this study, we analyzed associations among VM, ALDH1, KiSS-1, and MACC1 in EOC, and their respective correlations with clinicopathological characteristics and survival in EOC.

**Methods:**

Positive rates of VM, ALDH1, KiSS-1, and MACC1 in 207 whole EOC tissue samples were detected by immunohistochemistry. Patients’ clinical data were also collected.

**Results:**

Levels of VM, ALDH1, and MACC1 were significantly higher, and levels of KiSS-1 significantly lower, in EOC tissues than in benign ovary tumors. Levels of VM, ALDH1, KiSS-1, and MACC1 were associated significantly with tumor/lymph node/metastasis (LNM) grade, implantation, and International Federation of Gynecology and Obstetrics (FIGO) stage, and with patients’ overall survival (OS); whereas the KiSS-1+ subgroup had significantly longer OS than did the KiSS-1− subgroup. In multivariate analysis, high VM, ALDH1 or MACC1 levels, FIGO stage, implantation and low KiSS-1 levels were independently associated with shorter OS in patients with EOC.

**Conclusions:**

VM and expressions of ALDH1, KiSS-1, and MACC1 represent promising markers for metastasis and prognosis, and potential therapeutic targets for EOC.

## Background

In 2012, epithelial ovarian carcinoma (EOC) was reportedly found in 238,700 newly diagnosed cases of ovarian cancer (OC), and caused 15,1900 deaths [1], making it the fourth most lethal gynecological cancer [2]. The most commonly diagnosed OCs originate from epithelium; only a minority of OCs are from stromal or germ cells [3, 4]. As OC is usually asymptomatic in its early stages, >80% of patients diagnosed with OC in China have advanced-stage disease (e.g., with implantation or ascites). Despite advances in treatment, 5-year survival rates are still poor.

Although vasculogenesis and angiogenesis have been shown to promote tumor growth and metastasis, clinical benefits of anti-angiogenesis therapy for cancers are still unsatisfactory [5]. Treatments that address the other mechanism of tumor blood supply are urgently needed. Maniotis et al. reported a new blood supply phenomenon: vasculogenic mimicry (VM) which are channel-like structures formed by cancer cells [6]. Various highly aggressive cancer cells can mimic endothelial cells to form channel-like structures which could convey blood and nutrient, including gallbladder carcinoma [7], lung cancer [8], pancreatic cancer [9], glioblastoma [10], gastric carcinoma [11], hepatocellular carcinoma [12], and esophageal carcinoma [13]. VM is composed of highly aggressive tumor cells, rich extracellular matrix, and vessel-like structures. Patients with tumor-associated VM reportedly have poor prognoses and are prone to metastasis [6–13].

Tumor recurrence and metastasis may be related to cancer stem cells (CSCs), also named tumor-initiating cells, which have the ability to self-renew and initiate heterogeneous cancer cells that compose the tumors [14]. They make up a small part of the tumor cell population [14, 15], and are closely linked to slow proliferation rates and resistance to chemo- or radiotherapy [16]. CSCs can be isolated from various malignancies, using such markers as CD133, ALDH1, CD44, CD90, ABCG2, and CD117.

Aldehyde dehydrogenases (ALDHs) are found in the cytoplasm, nucleus, and mitochondria [17], and affect various fundamental biological process, such as proliferation, differentiation, survival, and oxidative stress [18]. ALDH1 is a common marker of CSCs, and a key member of ALDH superfamily. ALDH1 facilitates detoxification and metabolism of many endogenous and exogenous aldehydes, as well as synthesis of retinoic acid [19]. ALDH1 is a candidate biomarker for metastasis and prognosis of pancreatic carcinoma, gastric carcinoma, lung cancer, ovarian cancer, and breast cancer [17–21].

Inactivation of metastasis suppressor genes and activation of metastasis-promoting genes are early events in cancer invasiveness and metastasis. KiSS-1, a suppressor of metastasis, was first identified in melanoma, through analysis of subtractive hybridization [22]. It is located on chromosome 1q32 and encodes a 145-amino-acid protein that is cleaved into a family of KiSS peptins. KiSS-1 can bind to a G-protein-coupled receptor (GPR54 or KiSS-1R) and is believed to affect cell motility, invasion, proliferation, and metastasis [23]. Down-regulation (for example, through homozygous deletion, promoter methylation, or mutation) of KiSS-1 can increase tumor invasion and metastasis [24–26]. However, the precise function of KiSS-1 in tumor metastasis is still unclear. KiSS-1 is also been considered to be a useful marker of metastasis and prognosis.

Metastasis-associated in colon cancer 1 (MACC1) was the firstly identified by Stein et al. in colon cancer in 2009 [27]. MACC1 is a critical regulator of the hepatocyte growth factor/mesenchymal–epithelial transition (EMT) factor (HGF/c-Met) pathway and can control c-Met transcriptional activity by binding to the promoter of the c-Met gene [27, 28]. In vitro, MACC1 can activate the HGF/c-Met signaling pathway to induce EMT, which promotes tumor proliferation, invasion, and dissemination [29, 30]. In vivo, MACC1 can promote tumor growth and metastasis [27, 31]. MACC1 has been shown to affect recurrence, metastasis, and prognosis in various cancers [27–31].

Overall, studies of VM, ALDH1, KiSS-1, and MACC1 in association with tumor metastasis and prognosis suggest that these factors affect cancer progression; however, the associations among VM, ALDH1, KiSS-1, and MACC1 in EOC has not been widely reported. In this study, we examined the hypothesis that these factors are mutual correlated and are related to metastasis and prognosis in EOC.

## Methods

### Patients and tissue samples

We collected specimens from all 207 patients (median age: 59.1 years; range: 22–75 years) who were treated for EOC at the First Affiliated Hospital of Bengbu Medical College, from January 2008 to December 2010, along with 60 samples of benign tumors (serous- or mucinous-cystadenoma). Patients who had received preoperative chemo- or radiotherapy, or other anti-cancer therapy, were excluded. All tissue samples (including both benign and malignant tumors) were obtained with patients’ consent. This study was approved by the ethics committee of Bengbu Medical College and performed in accordance with the guidelines of the Declaration of Helsinki (No.BBMCEC2012063).. We included patients for whom we had complete pathological, clinical, and follow-up data (sampled at 6-month intervals by phone, mail, or e-mail). Overall survival (OS) was calculated from the patient’s surgery date to her death date or December 2015 (mean OS: 51.6 months; range: 6–95 months). As 20 patients had lost contact due to relocation, the cohort for survival data was 187 patients. Tumor-node-metastasis (TMN) stage was assessed according to the 2014^th^ edition of the International Federation of Gynecology and Obstetrics (FIGO). Tumors were graded according to World Health Organization standards (Table 1).

**Table 1:** Patients characteristics

Patients characteristics	Frequency (n)	Percentage (%)
Age (years)		
<60	126	60.9
≥60	81	39.1
Location		
Left	84	40.6
Right	81	39.1
Bilateral	42	20.3
Size (cm)		
<8.0	124	59.9
≥8.0	83	40.1
Type		
Serous	159	76.8
Mucinous	28	13.5
Endometrial	13	6.3
Clear cell	7	3.4
Ascite		
No	122	58.9
Yes	85	41.1
Grade		
Well	122	58.9
Moderate + poor	85	41.1
Implantation		
No	128	61.8
Yes	79	38.2
LNM		
No	129	62.3
Yes	78	37.7
FIGO stage		
I + II	106	51.2
III + IV	101	48.8

### Immunohistochemistry

Immunohistochemistry was performed according to the Elivision^TM^ Plus detection kit instructions (Lab Vision, USA). All EOC and control tissues were fixed in 10% buffered formalin and embedded in paraffin. Continuous 4-μm-thick sections were cut. All EOC and control sections were deparaffinized and dehydrated in xylene and graded alcohol, then washed with phosphate buffer saline (PBS, pH 7.2). Endogenous peroxidase activity was blocked by incubating sections in methanol containing 3% H2O2 for 10 min at room temperature (RT), then placed in citrate buffer (pH 6.0) and heated to 95 °C for antigen repair for 30 min. After several washes with PBS, all sections were quenched with goat serum at RT for 30 min, then incubated with mouse monoclonal antibody against human CD34 (Abcam, USA), ALDH1 (Abcam), KiSS-1 (Santa Cruz Biotechnology, Santa Cruz, CA, USA), or rabbit polyclonal antibody against human MACC1 (Santa Cruz Biotechnology) at 37 °C for 1 h. All samples were conducted periodic acid-Schiff (PAS)–CD34 dual staining to determine endothelial cells in glycosylated basement membranes of vessels, as well as vessel-like (VM) structure [8]. Furthermore, we found no necrosis and hemorrhage in tumor tissue near the VM structures.

A modified Yue’s method was used to assess VM in the EOC tissues and the control tissues [32]. All specimens were counterstained with hematoxylin, dehydrated, air-dried, and mounted. ALDH1+, MACC1+ and KiSS-1+ stains were all mainly seen in tumor cell cytoplasm.

### Evaluation of staining

Staining results were assessed semi-quantitatively by two independent pathologists who were blind to patients’ clinical and follow-up data. Ten high-power-fields (HPF) from different areas of each EOC slide were analyzed to avoid any intratumoral heterogeneity of antibody expression. Staining results were scored according to intensity (none: 0; weak: 1; moderate: 2; strong: 3) and extent (<11%: 1; 11–50%: 2; 51–75%: 3; >75%: 4). Intensity and extent scores were then multiplied to yield final scores that ranged 0–12. Scores ≥3 were considered positive. For sections that were positive for all three of ALDH1, KiSS-1, and MACC1, an average of the final score of each section was taken.

### Statistical analysis

Associations between clinicopathological characteristics and VM, ALDH1, KiSS-1, or MACC1 were compared using Fisher’s exact test or Chi-square test. Associations between VM, or ALDH1, or KiSS-1, or MACC1 was compared using Spearman’s coefficient test. Effects of VM, ALDH1, KiSS-1, or MACC1 on survival were determined by univariate and multivariate analyses. Independent prognostic factors were determined using the Cox regression model for multivariate analysis. The Kaplan–Meier method with log-rank test for univariate OS analysis was used to evaluate associations between VM+, ALDH1+, KiSS-1+, or MACC1+ results and clinicopathological characteristics, using SPSS 19.0 software for Windows (Chicago, IL). *P* < 0.05 was considered significant.

## Results

### Associations between VM, ALDH1, KiSS-1 and MACC1 expressions and clinicopathological characteristics

**Fig. 1:**
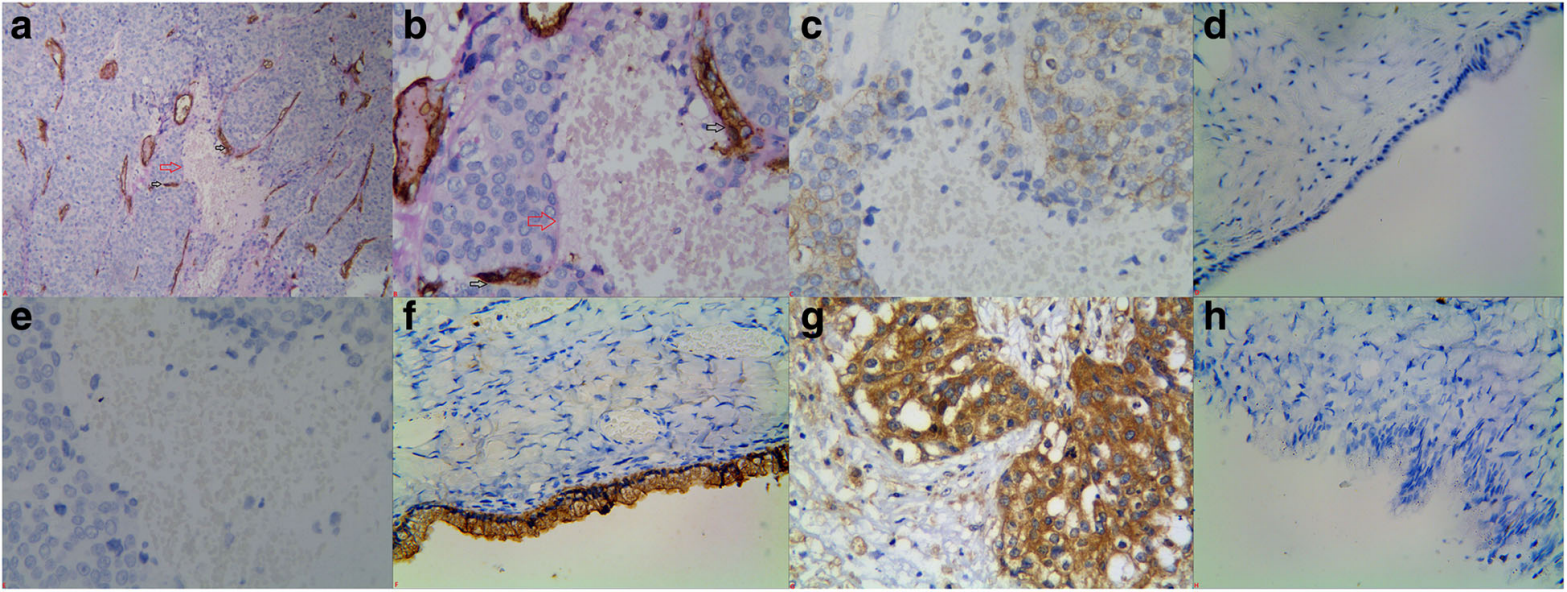
Positive staining of VM, or ALDH1, or KiSS-1, or MACC1 in epithelial ovarian carcinoma or the control tissue. **a** Positive staining of VM in the EOC tissue (400 magnification, *red arrow* is VM structure, *black arrow* is microvessel); **b** Positive staining of VM in the EOC tissue (400 magnification, *red arrow* is VM structure, *black arrow* is microvessel); **c** Positive staining of ALDH1 in the cytoplasm of cancer cells (400 magnification); **d** Negative staining of ALDH1 in the control tissue (100 magnification); **e** Negative staining of KiSS-1 in the EOC tissue (400 magnification); **f** Positive staining of KiSS-1 in the cytoplasm of the control cells (100 magnification); **g** Positive staining of MACC1 in the cytoplasm of cancer cells (400 magnification); **h**: Negative staining of MACC1 in the control tissue (100 magnification, **a**, **b**, **c**, **e** are serial sections)

To evaluate the contributions of VM, ALDH1, KiSS-1, and MACC1 to EOC, the results thereof were immunohistochemically evaluated for both EOC and control tissue samples. These data were then compared to patients’ clinicopathological characteristics. The rate of VM+ findings (small vessel, which is like a channel in EOC, the channel was PAS-positive but CD34-negative. The VM structure pattern included tubular, linear, and network, et al.) in the EOC samples (36.2%; 75/207) was significantly higher than that in the control tissues (0%; 0/60; *P* < 0.001; Fig. 1 a, b). VM in EOC was positively associated with tumor grade, LNM, implantation, and FIGO stage, but not patient age, tumor location, size, type, or ascites (Table 2).

**Table 2:** The correlation between VM, or ALDH1, or KiSS-1, orMACC1 and clinicopathological characteristics in epithelial ovarian carcinoma

Variable	VM		*P*	ALDH1		*P*	KiSS-1		*P*	MACC1		*P*
	Negative	Positive		Negative	Positive		Negative Positive			Negative	Positive	
Age (years)			0.690			0.664			0.855			0.839
<60	79	47		46	80		84	42		50	76	
≥60	53	28		32	49		53	28		31	50	
Location			0.727			0.737			0.504			0.422
Left	53	31		29	55		54	30		29	55	
Right	54	27		32	49		52	29		36	45	
Bilateral	25	17		17	25		31	11		16	26	
Size (cm)			0.570			0.614			0.749			0.879
<8.0	81	43		45	79		81	43		48	76	
≥8.0	51	32		33	50		56	27		33	50	
Type			0.155			0.144			0.856			0.238
Serous	97	62		55	104		107	52		61	98	
Mucinous	20	8		16	12		17	11		15	13	
Endometrial	8	5		5	8		9	4		3	10	
Clear cell	7	0		2	5		4	3		2	5	
Ascite			0.952			0.247			0.413			0.097
No	78	44		42	80		78	44		42	80	
Yes	54	31		36	49		59	26		39	46	
Grade			0.003			0.040			0.001			0.001
Well	88	34		53	69		70	52		59	63	
Moderate + poor	44	41		25	60		67	18		22	63	
Implantation			0.028			<0.001			0.001			<0.001
No	89	39		61	67		74	54		66	62	
Yes	43	36		17	62		63	16		15	64	
LNM			0.044			0.013			0.025			0.005
No	89	40		57	72		78	51		60	69	
Yes	43	35		21	57		59	19		21	57	
FIGO stage			<0.001			0.004			<0.001			<0.001
I + II	81	25		50	56		54	52		58	48	
III + IV	51	50		28	73		83	18		23	78	

As with VM, ALDH1+ expression was significantly higher in EOC tissues (62.3%, 129/207) than in control tissues (18.3%, 11/60; *P* < 0.001; Fig. 1 c, d). The rate of ALDH1+ expression in EOC was associated with tumor grade, LNM, implantation, and FIGO stage, but not patient age, tumor location, size, type, or ascites (Table 2).

KiSS-1+ expression was significantly less in EOC tissues (33.8%, 70/207) than in control tissues (96.7%, 58/60; *P* < 0.001; Fig. 1e, f). The rate of KiSS-1+ expression was inversely related to tumor grade, LNM, implantation, and FIGO stage. No association was found between KiSS-1 expression and patient age, tumor location, size, type, or ascites (Table 2).

MACC1+ expression was significantly higher in EOC tissues (60.9%, 126/207) than in control tissues (8.3%, 5/60; *P* < 0.001; Fig. 1 g, h). MACC1+ expression was also associated with tumor grade, LNM, implantation and FIGO stage, but not patient age, tumor location, size, type, or ascites (Table 2).

### Univariate and multivariate analyes

**Fig. 2:**
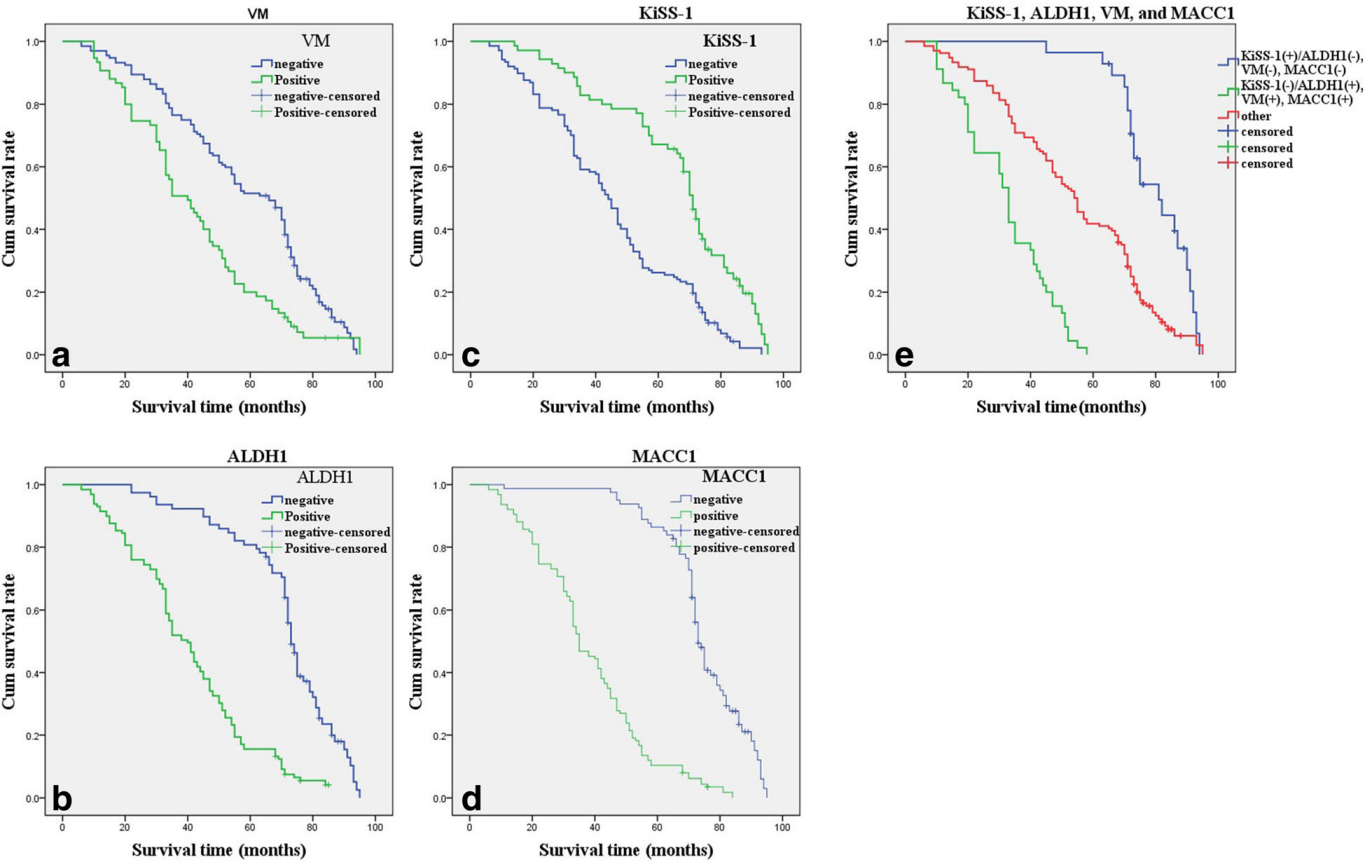
Kaplan-Meier analysis of the survival rate of patients with epithelial ovarian carcinoma. The y-axis represents the percentage of patients; the x-axis, their survival in months. **a** Overall survival of all patients in relation to VM (log-rank = 19.072, *P* < 0.001); **b** Overall survival of all patients in relation to ALDH1 expression (log-rank = 73.845, *P* < 0.001); **c** Overall survival of all patients in relation to KiSS-1 expression (log-rank = 29.001, *P* < 0.001); **d** Overall survival of all patients in relation to MACC1 expression (log-rank = 118.775, *P* < 0.001). In **a**, **b**, and **d** analyses, the green line represents patients with positive expression of VM, or ALDH1, or MACC1 with a trend of worse survival time than the blue line representing the negative VM, or ALDH1, or MACC1 group. In **d** analyses, the green line represents patients with positive expression of KiSS-1 with a trend of better survival time than the blue line representing the negative KiSS-1 group. **e** Overall survival of all patients in relation to the combination of KiSS-1, ALDH1, VM, and MACC1 expression (log-rank = 87.116, *P* < 0.001). The green line represents negative expression of KiSS-1 and positive expression of ALDH1, VM, MACC1 and the *blue line* represents positive expression of KiSS-1 and negative expression of ALDH1, VM, MACC1. The red line represents other positive or negative expression of the proteins

Follow-up data demonstrated that OS was significantly shorter in EOC patients with VM+ samples (41.6 ± 21.1 months) compared with those with VM− (57.3 ± 22.9 months; log-rank = 19.072, *P* < 0.00; Fig. 2a). Similarly, OS of ALDH1+ patients (40.4 ± 19.7 months) was significantly shorter than those of ALDH− patients (70.0 ± 16.7 months; log-rank = 73.845, *P* < 0.001; Fig. 2b). The OS of KiSS-1+ patients (64.6 ± 21.0 months) was significantly longer than those who were KiSS-1− (45.0 ± 21.9 months; log-rank = 29.001, *P* < 0.001; Fig. 2c). The OS of MACC1+ patients (38.0 ± 18.0 months) was significantly shorter than those who were MACC1− (72.7 ± 13.2 months; log-rank = 118.775, *P* < 0.001; Fig. 2d). The combination of KiSS-1− expression and ALDH1+, VM+, and MACC1+ expression led to poorer prognoses than did the reverse combination (log-rank = 87.116, *P* < 0.001; Fig. 2e). In univariate analysis, OS was significantly associated with clinicopathological characteristics, including grade (*P* = 0.001, log-rank = 14.060), LNM (*P* = 0.002, log-rank = 9.915), implantation (*P* < 0.001, log-rank = 68.810), and FIGO stage (*P* < 0.001, log-rank = 50.925; Table 3).

**Table 3:** Results of univariate analyses of overall survival time (OST)

Variable	n	Mean OS (months)	Log-rank	*P* value
VM			19.072	<0.001
Negative	132	57.3 ± 22.9		
Positive	75	41.6 ± 21.1		
ALDH1			73.845	<0.001
Negative	78	70.0 ± 16.7		
Positive	129	40.4 ± 19.7		
KiSS-1			29.001	<0.001
Negative	137	45.0 ± 21.9		
Positive	70	64.6 ± 21.0		
MACC1			118.775	<0.001
Negative	81	72.7 ± 13.2		
Positive	126	38.0 ± 18.0		
Age (years)			0.869	0.351
<60	126	52.8 ± 23.4		
≥60	81	50.0 ± 23.6		
Location			1.783	0.410
Left	84	46.9 ± 26.2		
Right	81	57.8 ± 17.4		
Bilateral	42	48.9 ± 25.7		
Size (cm)			3.386	0.066
<8.0	124	53.5 ± 24.2		
≥8.0	83	48.8 ± 23.3		
Type			6.662	0.083
Serous	159	51.1 ± 23.9		
Mucinous	28	60.5 ± 20.8		
Endometrial	13	43.5 ± 19.6		
Clear cell	7	42.3 ± 24.2		
Ascite			1.543	0.214
No	122	50.3 ± 22.7		
Yes	85	54.5 ± 24.6		
Grade			14.060	<0.001
Well	122	56.5 ± 23.9		
Moderate + poor	85	44.5 ± 21.0		
Implantation			68.810	<0.001
No	128	61.0 ± 21.2		
Yes	79	36.4 ± 18.6		
LNM			9.915	0.002
No	129	57.5 ± 21.6		
Yes	78	41.9 ± 23.3		
FIGO stage			50.925	<0.001
I + II	106	62.2 ± 22.1		
III + IV	101	40.5 ± 19.5		

Multivariate analysis indicated that VM+, ALDH1+, KiSS-1+, and/or MACC1+ specimens, and implantation and FIGO stage, were independent prognostic factors for EOC (Table 4).

**Table 4:** Results of multivariate analyses of overall survival time (OST)

Variable	B	SE	P	RR	95% CI
VM	0.408	0.170	0.017	1.503	1.076–2.100
ALDH1	0.621	0.241	0.010	1.860	1.160–2.983
KiSS-1	−0.371	0.181	0.041	0.690	0.484–0.985
MACC1	1.400	0.255	<0.001	4.057	2.460–6.691
Implantation	1.009	0.191	<0.001	2.744	1.886–3.992
FIGO stage	0.687	0.188	<0.001	1.987	1.376–2.871

### Associations among VM, and expression of ALDH1, KiSS-1, and MACC1 in EOC

Spearman correlation coefficient analysis showed negative associations between KiSS-1+ expression and that of VM (*r* = −0.284, *P* < 0.001), ALDH1 (*r* = −0.224, *P* = 0.001), or MACC1 (*r* = −0.306, *P* < 0.001). Expression of ALDH1 and that of VM (*r* = 0.150, *P* = 0.030) and MACC1 (*r* = 0.704, *P* < 0.001) were positively associated, as were VM and MACC1 (*r* = 0.193, *P* = 0.005; Table 5).

**Table 5:** Correlation among VM, ALDH1, KiSS-1, and MACC1 in EOC

Variable	VM		r	*P*	ALDH1		r	*P*	KiSS-1		r	*P*
	Negative	Positive			Negative	Positive			Negative	Positive		
MACC1			0.193	0.005			0.704	<0.001			−0.306	<0.001
Negative	61	20			65	16			39	42		
Positive	71	55			13	113			98	28		
VM							0.150	0.030			−0.284	<0.001
Negative					57	75			74	58		
Positive					21	54			63	12		
ALDH1			0.150	0.030							−0.224	0.001
Negative	57	21							41	37		
Positive	75	54							96	33		

## Discussion

EOC is a highly heterogeneous cancer, which can interfere with the reproducibility of biomarker evaluation. Therefore, prognostic value of candidate biomarkers must be thoroughly evaluated to ensure their validity. In this study, we found that VM was positively associated with tumor grade, LNM, implantation, and FIGO stage. Moreover, Kaplan–Meier survival analysis showed that VM+ EOC patients had significantly shorter OS than did VM− patients. These results suggest that VM plays a key role in EOC progression and metastasis, and could be a useful biomarker in managing this disease. VM may be responsible for the failure of anti-angiogenesis therapy [33–35]. Our results are consistent with previous studies, including those of OC and other cancers [8, 11, 12, 36].

ALDH1, an intracellular enzyme that helps detoxify and metabolize many endogenous and exogenous aldehydes, is commonly considered to be a CSC marker in various cancers [17–21]. Among the ALDH1 family subtypes, ALDH1A1 protects tumor cells against various cytotoxic drugs [37], whereas ALDH1A3 may play an important role in progression and metastasis; both are candidate biomarkers of prognosis in many cancer types [38]. ALDH1A3 was considered to be the isoenzyme responsible for ALDH activity and tumorigenicity in tumor cells [39]. In EOC, ALDH1 has been associated with poor prognosis [40] and poor response to chemotherapy [20]. The current study found that positive expression of ALDH1 may affect EOC development, invasion and metastasis, and is associated with poor prognosis. Some other studies showed similar results [17–21, 40].

KiSS-1 is widely regarded as a suppressor of tumor metastasis in various cancers [22–26]. KiSS-1 can inhibit cell motility, invasion, and metastasis [23] and reduce metastatic potential; however, it does not inhibit tumorigenicity [25]. Findings in this study also demonstrated that KiSS-1 expression was significantly lower in EOC tissues than in control tissues, and its expression was inversely associated with tumor grade, LNM, implantation and FIGO stage. In addition, Kaplan- Meier survival indicated that EOC patients with KiSS-1+ specimens had significantly higher survival rates than did KiSS-1− patients. These results suggest that KiSS-1 down-regulation promotes EOC progression and metastasis, which is similar to results of previous studies [22–26, 41].

MACC1 is a key regulator of the HGF/c-Met pathway which reportedly affects tumorigenicity, epithelial–mesenchymal transition (EMT), cell motility, invasion, and metastasis [27, 28, 42]. The current study found that MACC1 expression in EOC tissues was significantly higher than in control tissues. We also found MACC1 overexpression to be positively related to tumor grade, LNM, implantation, and FIGO stage. As with ALDH1, OS of MACC1+ EOC patients was significantly shorter that for the MACC1− subgroup. Our results are similar to previous studies of EOC [43, 44], which suggests that MACC1 could be a useful biomarker for EOC.

FIGO staging guides therapeutic strategies for patients with EOC, but does not provide clear information about EOC’s behavior. Therefore, novel and effective biomarkers to predict EOC behavior, metastasis, and patient prognosis are urgently needed. In this study, multivariate Cox regression analysis indicated that VM, ALDH1, KiSS-1, and MACC1 expression and implantation, as well as FIGO stage, are independent prognostic factors for EOC patients. Our results thus support VM, ALDH1, KiSS-1, and MACC1 as credible biomarkers for EOC, especially in predicting metastasis and prognosis.

Moreover, as ALDH1 is a marker for CSCs, its involvement in CSCs may play an important role in the initiation and progression of EOC. CSCs can induce angiogenesis to provide adequate nutrition and oxygen for rapid tumor growth [45], and can apparently differentiate tumor cells and endothelial cells [46]; thus CSCs can mimic endothelial cells to form VM structures in the host microcirculation system. EMT plays a key role in tumorigenesis and in VM [47–49]. As MACC1 regulates the HGF/c-Met signaling pathway, it can promote VM [11]. KiSS-1 can inhibit NF-κB binding to the MMP9 promoter [50] which degrades the extracellular matrix; whereas decreased KiSS-1 should down-regulate its inhibition of metastasis, thus further promoting invasion and metastasis.

## Conclusions

Our results imply that ALDH1 affects EOC evolution; and that combined detection of VM, ALDH1, KiSS-1, and MACC1 are valuable indicators of metastasis and prognosis in EOC.

## Abbreviation

ALDH1: Aldehyde dehydrogenase 1
CSC: Cancer stem cells
EMT: Epithelial-mesenchymal transition
EOC: Epithelial ovarian carcinoma
FIGO: International Federation of Gynecology and Obstetrics
HGF/c-Met: Hepatocyte growth factor/mesenchymal-epithelial transition
HPF: High power field
LNM: Lymph node metastasis
MACC1: Metastasis-associated in colon cancer 1
OC: ovarian carcinoma
OST: overall survival time
PAS: periodic acid-Schiff
PBS: phosphate buffer saline
RT: room temperature
TIC: tumor initiating cells
VM: vasculogenic mimicry
WHO: World Health Organization
